# Betaine affects muscle lipid metabolism via regulating the fatty acid uptake and oxidation in finishing pig

**DOI:** 10.1186/s40104-017-0200-6

**Published:** 2017-09-01

**Authors:** Sisi Li, Haichao Wang, Xinxia Wang, Yizhen Wang, Jie Feng

**Affiliations:** Key Laboratory of Animal Nutrition & Feed, Zhejiang Province, College of Animal Science, Zhejiang University, Hangzhou, China

**Keywords:** Betaine, Fatty acid intake, Fatty acid oxidation, Muscle, Pig

## Abstract

**Background:**

Betaine affects fat metabolism in animals, but the specific mechanism is still not clear. The purpose of this study was to investigate possible mechanisms of betaine in altering lipid metabolism in muscle tissue in finishing pigs.

**Methods:**

A total of 120 crossbred gilts (Landrace × Yorkshire × Duroc) with an average initial body weight of 70.1 kg were randomly allotted to three dietary treatments. The treatments included a corn–soybean meal basal diet supplemented with 0, 1250 or 2500 mg/kg betaine. The feeding experiment lasted 42 d.

**Results:**

Betaine addition to the diet significantly increased the concentration of free fatty acids (FFA) in muscle (*P* < 0.05). Furthermore, the levels of serum cholesterol and high-density lipoprotein cholesterol were decreased (*P* < 0.05) and total cholesterol content was increased in muscle (*P* < 0.05) of betaine fed pigs. Experiments on genes involved in fatty acid transport showed that betaine increased expression of *lipoprotein lipase(LPL)*, *fatty acid translocase/cluster of differentiation* (*FAT/CD36*), *fatty acid binding protein* (*FABP3*) and *fatty acid transport protein* (*FATP1*) (*P* < 0.05). The abundance of fatty acid transport protein and fatty acid binding protein were also increased by betaine (*P* < 0.05). As for the key factors involved in fatty acid oxidation, although betaine supplementation didn’t affect the level of carnitine and malonyl-CoA, betaine increased mRNA and protein abundance of carnitine palmitransferase-1(CPT1) and phosphorylated-AMPK (*P* < 0.05).

**Conclusions:**

The results suggested that betaine may promoted muscle fatty acid uptake via up-regulating the genes related to fatty acid transporter including *FAT/CD36*, *FATP1* and *FABP3*. On the other hand, betaine activated AMPK and up-regulated genes related to fatty acid oxidation including *PPARα* and *CPT1*. The underlying mechanism regulating fatty acid metabolism in pigs supplemented with betaine is associated with the up-regulation of genes involved in fatty acid transport and fatty acid oxidation.

## Background

Betaine is a derivative of the amino acid glycine with three chemically reactive methyl groups. Betaine is distributed widely in animals, plants and microorganisms, and it is also a metabolite of choline oxidation in animals [1]. The principal physiologic role of betaine is as a methyl group donor [2], which means betaine participates in many important biochemical pathways, including methionine-homocysteine cycle and the biosynthesis of many compounds such as carnitine, creatine and phospholipids. Since carnitine is required for transport of long chain fatty acids into mitochondria [3], scientists have paid much attention to effects of betaine on energy metabolism especially lipid metabolism in animals. Studies showed that dietary betaine supplementation affected energy partitioning in pigs [4, 5] and it’s also widely reported that betaine promotes animal growth and decreases carcass fat percentage in finishing pigs [6–10]. Further investigations found that betaine supplementation could decrease hepatic triglyceride accumulation [11, 12] and prevent fatty liver in rats fed high-fat-diets [13, 14]. The intramuscular fat content in the longissimus muscle was increased when pigs were fed betaine [15, 16]. Madeira et al. [17] reported that betaine might be involved in the differential regulation of some key genes of lipid metabolism in muscle and subcutaneous adipose tissue. However, studies on the mechanism of betaine affecting lipid metabolism in muscle are lacking. Therefore, the objective of the present study was to investigate possible mechanisms of betaine in altering lipid metabolism in muscle tissue of finishing pigs.

## Methods

### Animals and treatments

The experiment protocol used in this study was approved by the Institutional Animal Care and Use Committee of Zhejiang University. A total of 120 crossbred gilts (Landrace × Yorkshire × Duroc) with an average initial body weight of 70.1 kg (SD 0.70 kg) were randomly allotted to three dietary treatments. Each treatment consisted of four pens replicates with 10 gilts per pen. The treatment diets included a corn–soybean meal basal (Table 1) supplemented with 0, 1250 mg/kg (Low Betaine) or 2500 mg/kg (High Betaine) betaine (provided by Healthy Husbandry Sci-tech Co., Ltd. Hangzhou, China) respectively at the expense of corn. The basal diet was formulated to meet or exceed the nutrient requirements of finishing pigs [18]. Chemical analyses of the basal diet were carried out according to the methods of AOAC [19]. The feeding experiment lasted 42 d after a 7-day adaptation period. All pigs were housed in a curtain-sided pig barn with concrete slotted floors. Feed and water were provided for ad libitum consumption throughout the experiment.

**Table 1:** Nutrition formulation of basic diet

Ingredients	%	Nutrient	%
Corn	67.83	Digestible energy, MJ/kg^a^	13.42
Soybean meal	23	Dry matter	87.09
Rapeseed meal	3	Crude protein	17.02
Wheat midding	3	Crude fat	3.98
CaHPO_4_	1.5	Calcium	0.85
Limestone	1.0	Phosphorus	0.64
Salt	0.3	Lysine	0.92
Lysine	0.10	Met	0.27
Trace element premix^b^	0.25		
Vintamin premix^c^	0.02		

^a^All of the data were analyzed value except digestible energy which was calculated using swine NRC(2012) values

^b^Provided the following amounts per kilogram of diet: Fe (FeSO4·7H_2_O), 50 mg; Cu (CuSO4·5H_2_O), 5 mg; Mn (MnSO4·H_2_O), 5 mg; Zn (ZnSO4·7H_2_O), 50 mg; I (KI), 0.35 mg; Se (NaSe_2_O_3_), 0.15 mg

^c^Provided the following amounts per kilogram of diet: vitamin A, 3000 IU; vitamin D_3_, 610 IU; vitamin E, 20 IU; vitamin B_2_, 5 mg; vitamin B_12_, 0.021 mg; biotin, 0.1 mg; pantothenic acid, 10 mg; nicotinic acid, 15 mg

### Sample collection

At the end of the trial, eighteen pigs (six from each dietary treatment) weighing about 111.8 kg (SD 2.08 kg) were selected to collect tissue samples. Following an overnight fast, pigs were stunned by electrical shock and bleeding. Individual blood samples were collected at slaughter during exsanguinations. After collection of blood, samples were kept at room temperature for 2 h and then centrifuged for 10 min at 3000×g at 4 °C. Serum was collected and frozen at −80 °C until subsequent analyses. Samples of longissimus muscle between the 6^th^ and 7^th^ rib were obtained on the left side of the carcass within 5 min after slaughter, and then snap frozen in liquid nitrogen and stored at −80 °C until subsequent analyses.

### Analysis of lipid metabolites in serum

Serum concentration of high-density lipoprotein cholesterol (HDLC), total cholesterol (TC), free fatty acid (FFA) and triglyceride were measured with commercial assay kits (Nanjing Jiancheng Bio-engineering Institute, Code No. A112–2, A111–2, A042–1 and A110–2, respectively, Nanjing, China) following the manufacturer’s instructions.

### Muscle lipid metabolites analysis

A 10% muscle homogenate was prepared with a mixture of chloroform and formaldehyde (a volume ratio of 2:1). Then extracted at room temperature for 24 h [20]. The organic solvent layer was taken and the level of triglyceride in muscle was measured with commercial assay kit (Nanjing Jiancheng Bio-engineering Institute, A110–2, Nanjing, China). Before the levels of TC and FFA in muscle were measured by the kits (Nanjing Jiancheng Bio-engineering Institute, Code No. A112–2, A111–2, A042–1 and A110–2, respectively, Nanjing, China), muscle tissue was made homogenate with physiological saline. The concentrations of carnitine and malonyl-CoA were measured using ELISA kits (Biovol Technologies, Code No.50R–E.3088P & 50R–E.3035P, Shanghai, China) for porcine assay according to the instructions.

### RT-PCR analysis

Total RNA was extracted from frozen porcine muscle tissue using the Trizol reagent as described by the manufacturer (Invitrogen). The RNA concentration and purity were determined by the NanoDrop ND-2000 spectrophotometer (Thermofisher, USA) and its integrity was confirmed by agarose gel electrophoresis. The cDNA synthesis was performed in a 10-μL reaction volume containing 2 μg total RNA using the SYBR PrimeScript™ RT-PCR kit with gDNA Eraser (Code No. RR047A, TaKaRa, Dalian, China). Genomic DNA is eliminated by treatment for 2 min at 42 °C with gDNA Eraser, which has potent DNA degrading activity. Then a reverse-transcription reaction reagent is added that includes a component that completely inhibits DNA degradation activity, and the reverse-transcription reaction proceeds for 15 min at 37 °C. The abundance of the target genes was measured by quantitative real-time PCR, performed with the ABI Stepone Plus™ RT-PCR system (ABI Biotechnology, USA) using SYBR Premix Ex Taq™ (Tli RNaseH Plus) RT-RCR kit (TaKaRa, Dalian, China). Primers for the selected genes were synthesized commercially by Invitrogen (Shanghai, China), shown in Table 2. The reaction protocol comprised a cycle of 95 °C for 1 min, 40 cycles of 95 °C for 10 s and 64 °C for 25 s. The expression of the target genes were normalized by the endogenous housekeeping gene (β-actin) [21, 22]. Each sample was analyzed in triplicate and the PCR amplification efficiency was close to 100%. The gene expression was calculated by using the comparative (2 ^− ΔΔCt^) method [23].

**Table 2:** Primers of target genes for RT-PCR

Genes	GenBank accession	Primers sequences(5′ to 3′)	Product size, bp	Annealing temperature, °C
*β-actin*	XM_003124280.3	CCTGCGGCATCCACGAAAC	123	63
		TGTCGGCGATGCCTGGGTA		
*AMPKα2*	AY159788.1	GGTCTGGTTCCTCAACACCTCA	90	63
		GGCTCTCCGCAGTGACAGAAT		
*PPARγ*	NM_214379	GTGGAGACCGCCCAGGTTTG	108	64
		GGGAGGACTCTGGGTGGTTCA		
*LPL*	NM_214286.1	CCCTATACAAGAGGGAACCGGAT	138	63
		CCGCCATCCAGTCGATAAACGT		
*CPT1*	NM_001007191.1	GGACGAGGAGTCTCACCACTATGAC	128	63
		TCTTGAACGCGATGAGGGTGA		
*FATP1*	NM_001083931.1	CCCTCTGCGTCGCTTTGATG	151	63
		GCTGCGGTCCCGGAAATACA		
*FAT/CD36*	NM_001044622.1	CTGGTGCTGTCATTGGAGCAGT	160	63
		CTGTCTGTAAACTTCCGTGCCTGTT		
*FABP3*	NM_001099931.1	CCAACATGACCAAGCCTACCACA	176	63
		ACAAGTTTGCCTCCATCCAGTGT		
*PPARα*	NM_001044526.1	GGCTTACGGCAATGGCTTCA		
		CGGTCTCCGCACCAAATGA	168	64

### Western blot analysis

Protein form muscle samples was extracted by T-PER Tissue Protein Extraction Reagent containing protease inhibitor cocktail (Thermo Pierce, Code No.78510, USA), and quantified with BCA protein assay kit (Beyotime, Code No.P0010, Shanghai, China) according to kit instructions. Proteins were separated on SDS - PAGE gels (12%), and then electrophoretically transferred onto immobilon-P polyvinylidene fluoride membranes (PVDF membrane, Millipore, Code No. IPVH00010, America). Membranes were blocked 1 h in Tris-buffered saline containing 5% nonfat-dried milk at room temperature. Membranes were then incubated overnight at 4 °C in blocking buffer containing primary antibodies (as shown in Table 3). A goat anti-rabbit IgG (H + L) Secondary antibody (Thermo Pierce, Code NO.31210, USA) with 1/5000 dilution was used in the detection of specific proteins. For loading control, β-actin antibody was used as control. In addition, the relative expression of p-AMPK was normalized with AMPK. Finally, Super Signal West Dura Extended Duration Substrate (Thermo Pierce, Code No. 34075, USA) was used to visualize the protein bands. Band intensities were determined by using BandScan 5.0 software.

**Table 3:** The primary antibodies for Western blot

Primary antibody	Order numbers	Dilution	Size, kDa
Anti-Cardiac FABP	abca ab45966	1:1500	15
Anti-FATP1	abcam ab81875	1:2000	65
Anti-CPT1B	abcam ab104662	1:2000	88
Anti-Phospho-AMPK	Cell Signaling Technology 2535	1:1000	62
Anti-AMPKα	Cell Signaling Technology 5832	1:1000	62
β-actin (C4)	Santa Cruz SC-47778	1:1500	43

The relative expressions of target proteins = (The optical density of target proteins/The optical density of β-actin).

### Statistical analyses

Results were presented as means and standard deviations. Statistical analysis was performed by one-way analysis of variance (ANOVA) and the Duncan method was used to put up multiple comparison with the statistical software SPSS 19.0. In all analyses, the level of significant difference was set at *P* < 0.05.

## Results

### Betaine on serum lipid metabolites

**Fig. 1:**
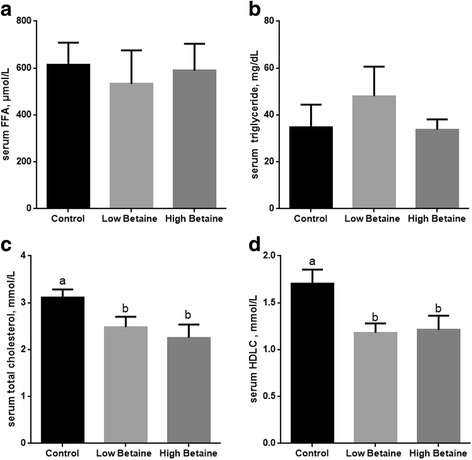
Effect of betaine supplementation on serum parameters of lipid metabolism. The levels of serum free fatty acid (FFA, **a**), triglyceride (**b**), total cholesterol (**c**) and high-density lipoprotein cholesterol (HDLC, **d**). ^a,b^Values without common superscript letters differ significantly (*P* < 0.05). Low betaine and high betaine represent 1250 mg/kg and 2500 mg/kg betaine addition, respectively

As shown in Fig. 1, there was no significant difference in the levels of serum FFA and triglyceride in the pigs fed betaine compared with control group. Additionally, the concentration of HDLC and TC were significantly lower in the betaine treated pigs (*P* < 0.05).

### Betaine on muscle lipid metabolites

**Fig. 2:**
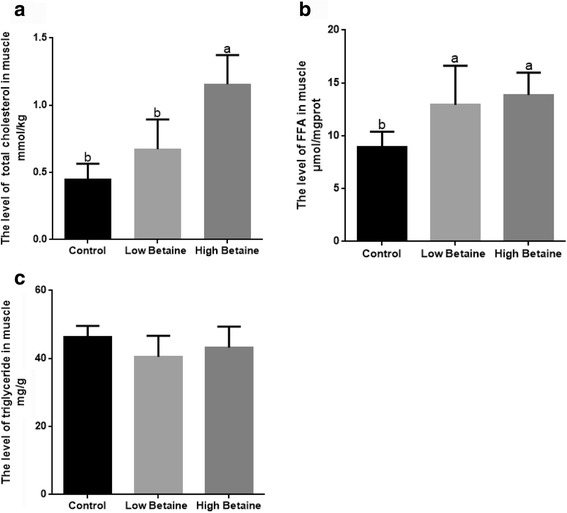
Effect of betaine supplementation on total cholesterol, FFA and triglyceride in muscle. The levels of total cholesterol (**a**), free fatty acid (FFA, **b**) and triglyceride (**c**) in muscle. ^a,b^Values without common superscript letters differ significantly (*P* < 0.05). Low betaine and high betaine represent 1250 mg/kg and 2500 mg/kg betaine addition, respectively

The level of FFA and TC were markedly higher in muscle when pigs were fed betaine (*P* < 0.05*,* Fig. 2). Compared to the control group, the level of triglyceride in muscle was not affected by betaine addition (*P* > 0.05).

### Key factors involved in muscle FFA intake

**Fig. 3:**
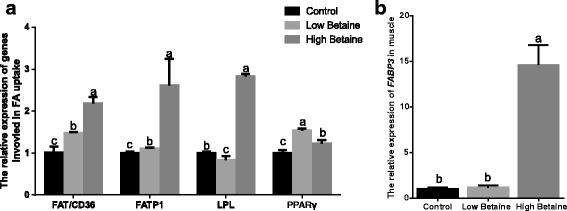
The relative gene expression of key factors involved fatty acid uptake in muscle. mRNA expression was performed by RT-PCR and β-actin was chosen as reference gene. **a**The relative expression of *FAT/CD36, FATP1, LPL* and *PPARγ* in muscle, (**b**) The relative expression of *FABP3* in muscle. ^a,b^Values without common superscript letters differ significantly (*P* < 0.05). Low betaine and high betaine represent 1250 mg/kg and 2500 mg/kg betaine addition, respectively

**Fig. 4:**
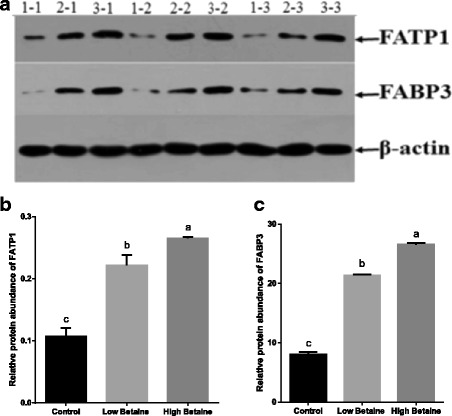
The relative protein abundance of FATP1 and FABP3 in muscle. Western blot results were shown in **a** (The control group: 1–1, 1–2, 1–3; Low betaine group: 2–1, 2–2, 2–3; High betaine group: 3–1, 3–2, 3–3). Data were normalized with β-actin as shown in **b**, **c**. ^a,b^Values without common superscript letters differ significantly (*P* < 0.05). Low betaine and high betaine represent 1250 mg/kg and 2500 mg/kg betaine addition, respectively

As shown in Fig. 3, the gene expression of *FAT/CD36*, *FATP1* and *PPARγ* (*P* < 0.05) were higher in betaine-fed groups than control group. The addition of 2500 mg/kg betaine markedly up-regulated the gene expression of *FABP3* and *LPL* (*P* < 0.05). In addition, the abundance of fatty acid transport protein and fatty acid binding protein were significantly increased by betaine supplementation (*P* < 0.05, Fig. 4).

### Key factors involved in muscle FA oxidation

**Fig. 5:**
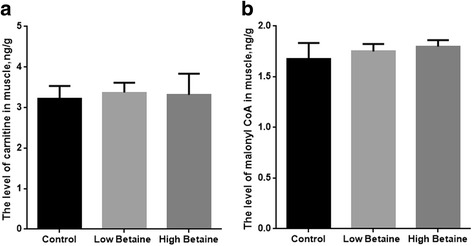
Effect of betaine supplementation on the level of carnitine(**a**) and malonyl-CoA (**b**) in muscle. Low betaine and high betaine represent 1250 mg/kg and 2500 mg/kg betaine addition, respectively

Betaine supplementation did not affect carnitine or malonyl-CoA in muscle compared to the control group (*P* > 0.05, Fig. 5).

**Fig. 6:**
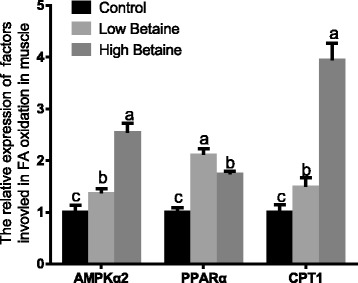
The relative mRNA expression of factors involved in fatty acid oxidation in muscle. mRNA expression was performed by RT-PCR and β-actin was chosen as reference gene. ^a,b^Values without common superscript letters differ significantly (*P* < 0.05). Low betaine and high betaine represent 1250 mg/kg and 2500 mg/kg betaine addition, respectively

**Fig. 7:**
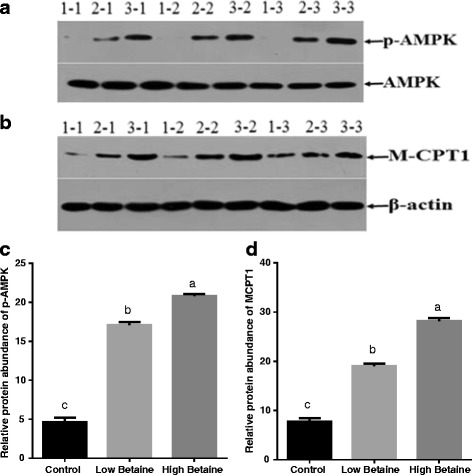
The relative protein abundance of p-AMPK and M-CPT1. The results of western blot were showed **a** and **b** (The control group: 1–1, 1–2, 1–3; Low betaine group: 2–1, 2–2, 2–3; High betaine group: 3–1, 3–2, 3–3). p-AMPK (the activated form of AMPK) was normalized with AMPK (shown in **c**) and MCPT1(the muscle type of CPT1) was normalized with β-actin (shown in **d**). ^a,b^Values without common superscript letters differ significantly (*P* < 0.05). Low betaine and high betaine represent 1250 mg/kg and 2500 mg/kg betaine addition, respectively

The gene expression of *AMPKα2*, *PPARα* and *CPT1* were significantly higher in pigs fed with betaine than the control group. (*P* < 0.05*,* Fig. 6). Furthermore, betaine supplementation markedly increased the abundance of phosphorylated-AMPK and CPT1 in muscle (*P* < 0.05, Fig. 7).

## Discussion

Fatty acid metabolism in muscle includes uptake, synthesis and oxidation [24–26], but the synthesis is at a slow rate [27]. The main source of fatty acid in muscle tissue includes transport from plasma and hydrolysis from chylomicron and very-low-density-lipoprotein (VLDL) with LPL. Our study found that the concentration of FFA was significantly increased in muscle when pigs were fed betaine, similarly to the studies carried out by Yang et al. [28] and Fernández-Fígares et al. [29]. We speculated that the transport of FFA and/or the hydrolysis may be enhanced. More experiments were carried out regarding factors involved in fatty acid transport in muscle tissue. It is widely recognized that long chain fatty acid (LCFA) cross the plasma membrane via a protein-mediated mechanism. A number of fatty acid transporters have been identified, including fatty acid translocase/cluster of differentiation (FAT/CD36) and fatty acid transport proteins (FATP1) [30]. We found that betaine supplementation up-regulated gene expression for *FATP1* and *FAT/CD36*. Experiments in vitro have shown that over expression of *FATP1* increased the uptake of LCFA in cells [31] and studies in vivo documented that muscle-specific over-expression of *FAT/CD36* enhanced cellular fatty acid uptake in mice [32]. FABP3, another important protein in fatty acid transportation, plays a role in transporting fatty acid from the sarcolemma to their intracellular sites of metabolism [33]. In muscle cells, the intracellular transport of LCFAs is facilitated to a great extent by FABP3 [34] Additionally, FABP3 is confirmed to be associated with intramuscular fat in pigs [35]. Our studies showed that feeding betaine up-regulated the protein abundance of FABP3. In addition, the gene expression of *FABP3* was enhanced when pigs were fed with 2500 mg/kg betaine but no difference was found with 1250 mg/kg-betaine addition. The possible reason maybe that *FABP3* expression is translationally rather than transcriptionally regulated [36]. In summary, betaine may promote the uptake of fatty acids in muscle via regulating the expression of *FAT/CD36*, *FATP1* and *FABP3*. As mentioned above, LPL is the principal enzyme that hydrolyzes circulating triglycerides and it also can increase lipid uptake [37]. The results showed a significant increase in the gene expression of *LPL* with the addition of 2500 mg/kg betaine, which indicates betaine might enhance lipid uptake as well as chylomicron hydrolysis. The nuclear receptor PPARγ is a central regulator of adipose tissue development and an important modulator of expression in adipocytes [38]. To date, only a limited number of genes are known to be direct targets of PPARγ in adipose tissue. The majority of these encode proteins with direct links to lipid metabolism including LPL, FATP and FAT/CD36 [39, 40]. In present study, the gene expression of *PPARγ* was significantly higher in betaine-fed groups than the control group. We found that the effect of betaine on PPARγ was similar to its downstream target genes. All these results were similar to Albuquerque [41] and imply that betaine may facilitate fatty acids uptake in muscle via affecting key factors involved in FFA uptake, and the specific regulation mechanism needs more research.

The concentration of FFA in muscle tissue resultes from the balance of transport and oxidation. As a methyl donor, betaine participates in the biosynthesis of carnitine and because of this, betaine may be related to fatty acid β-oxidation. LCFAs are first transformed into acyl CoA, then transferred into mitochondria after combining with carnitine where it is oxidized. Carnitine palmitoyl transferase I (CPT1) is the rate-limiting enzyme that controls the step of combination and malony-CoA is an allosteric inhibitor of CPT1 [42]. Whereas the synthesis of malonyl-CoA is catalyzed by acetyl-CoA carboxylase (ACC), the activity of the ACC is regulated by phosphorylation of AMPK [43]. Hence, AMPK-ACC-CPT1 is an important signaling pathway to regulate fatty acid β-oxidation in mitochondria. Cai et al. [44] found that gestational dietary betaine supplementation down-regulated expression of *ACC* in neonatal piglets and Pekkinen et al. [11] found betaine supplementation had an impact on carnitine metabolism in high-fat-fed mice. Our experiment didn’t find significant changes in muscle concentrations of malonyl-CoA or carnitine. The different results might be related to the different experiment condition and the mechanism needs to be further investigated. Increased gene expression and protein expression of *CPT1* were up-regulated with betaine addition, which implied betaine may enhance fatty acid β-oxidation in muscle tissue. However, others have shown betaine supplementation reduced the activity of CPT1 and mRNA abundance, and further increased IMF in finishing pigs [Duroc × (Seghers × Seghers)] [15]. We speculate that the effect of betaine addition on CPT1 might be influenced by breed and muscle type. In order to get a better understanding, we further analyzed effects of betaine on AMP-activated protein kinase (AMPK) and PPARα, which are both upstream regulatory factors of CPT1. AMPK is a crucial energy sensor for cells, which can promote the catabolism of fatty acids by enhancing their uptake into mitochondria and their consequent breakdown by beta-oxidation [45]. It was reported that activated AMPK in muscle enhances the gene expression of *PPARα* and *CPT1* [46], and CPT1 also seems to be a target of PPARα [47]. In the current experiment, the gene expression of both *PPARα* and *AMPK* were higher in betaine-fed groups as well as protein expression of p-AMPK (the activated form of AMPK). Similar to our previous report in rat liver [12], it can be inferred that betaine affected fatty acid oxidation in muscle via activating AMPK and up-regulated *PPARα* and *CPT1* gene expression.

The effect of betaine supplementation on cholesterol metabolism was of interest. The present study showed that betaine supplementation decreased the concentration of serum cholesterol and HDLC and increased cholesterol level in muscle, which was consistent with the studies by Albuquerque et al. [41] and Yang et al. [20]. However, Matthews et al. [48] and Martins et al. [49] reported that betaine supplemented pigs presented higher serum cholesterol. The efficacy of betaine in regulating the concentration of cholesterol in pigs shows variable results and seems to depend on both animal and dietary factors. Although the results were inconsistent, it seems to indicate that betaine might affect cholesterol partitioning or maybe enhances the transport of cholesterol in pigs, and more research is needed to clarify the specific mechanism.

## Conclusions

In present study, betaine supplementation increased the level of free fatty acids in muscle, which may have resulted due to a change in the balance of fatty acid uptake and oxidation. Betaine may promote fatty acid uptake via increasing the expression of fatty acid transporters including FAT/CD36, FATP1 and FABP3 in muscle. Additionally, betaine activated AMPK and up-regulated *PPARα* and *CPT1*, and may enhance fatty acid oxidation in muscle. Fatty acid accretion in muscle represents a balance between uptake and oxidation, and it seems that the effect of betaine on uptake was stronger than oxidation.

## Abbreviations

CPT1: Carnitine palmitoyl transferase 1
FABP3: Fatty acid binding protein
FAT/CD36: Fatty acid translocase/cluster of differentiation
FATP1: Fatty acid transporter protein1
FFA: Free fatty acid
HDLC: High-density lipoprotein cholesterol
LPL: Lipoprotein lipase
TC: Total cholesterol
